# ANGPTL2 and CP define complementary circadian-associated signals in atrial fibrillation: integrative transcriptomics and external validation

**DOI:** 10.3389/fcell.2026.1949794

**Published:** 2026-09-21

**Authors:** Jiahui Wang, Jintao Qian, Junhao Zhang, Guoyu Cao, Boyang Chen, Jian Shi, Qing Zhou

**Affiliations:** 1 Department of Cardiothoracic Surgery, Nanjing Drum Tower Hospital, Clinical College of Nanjing University of Chinese Medicine, Nanjing, Jiangsu, China; 2 Department of Cardiothoracic Surgery, Nanjing Drum Tower Hospital, Affiliated Hospital of Medical School, Nanjing University, Nanjing, China; 3 Department of Cardiothoracic Surgery, Affiliated Hospital of Putian University, Putian, Fujian, China

**Keywords:** ANGPTL2, atrial fibrillation, atrial transcriptomics, ceruloplasmin, circadian rhythm, external validation, single-nucleus RNA sequencing

## Abstract

**Introduction:**

Atrial fibrillation (AF) develops through the interplay of electrical, structural, inflammatory, and metabolic remodeling. However, the circadian-associated molecular characteristics of the diseased human atrium remain incompletely understood.

**Methods:**

We integrated differential expression analysis, circadian gene intersection, weighted gene co-expression network analysis, and complementary feature selection algorithms in a discovery cohort. The prioritized candidate genes were subsequently validated using an independent atrial dataset, human atrial rhythmicity data, single-nucleus transcriptomics, and experimental evidence derived from human and mouse atrial tissues. We additionally performed cross-cohort drug repurposing screening and exploratory molecular docking.

**Results:**

ANGPTL2 and CP were identified as prioritized candidate genes associated with circadian rhythms. Both genes were consistently upregulated in fibrillating atria but exhibited distinct expression profiles: ANGPTL2 demonstrated stronger discriminatory power at the tissue level, whereas CP showed a larger but more heterogeneous expression alteration. Both genes displayed rhythmic expression patterns in human atrial appendage tissue and were predominantly enriched in vascular and stromal cell populations, establishing a link between temporal atrial biology and inflammatory-metabolic remodeling as well as copper-redox remodeling. The upregulation of their transcripts and proteins was further validated across independent human samples and an arrhythmia-prone mouse model. No compound met the prespecified multi-source criteria for cross-cohort drug repurposing. An independent exploratory analysis of clinically used antiarrhythmic drugs identified favorable molecular docking profiles for both target proteins.

**Discussion:**

ANGPTL2 and CP are externally validated circadian rhythm-associated candidate genes for atrial fibrillation (AF), and may yield experimentally testable candidate gene-drug pairs. However, these findings do not establish causal regulation by the circadian clock, direct molecular binding, or clinical therapeutic efficacy.

## Introduction

Atrial fibrillation (AF) is the most prevalent sustained arrhythmia globally and is associated with elevated risks of stroke, heart failure, impaired quality of life, and premature mortality (Cheng et al., 2024; Elliott et al., 2023; Sagris et al., 2021). AF persistence is driven by the interactive effects of electrical, structural, inflammatory, metabolic, and autonomic remodeling, rather than a single molecular abnormality (Allessie, 2002; Bode et al., 2024; Dobrev et al., 2023; Goette et al., 2024; Vinciguerra et al., 2024). These processes progressively remodel the atrial substrate, which accounts for the high rate of AF recurrence even after initially successful rhythm control interventions. Identification of molecular signals that capture reproducible characteristics of this remodeling could improve mechanistic stratification of the disease and inform more targeted experimental investigations.

The circadian system represents a biologically plausible yet understudied regulatory layer in AF pathophysiology. Central and peripheral circadian clocks coordinate diurnal variations in cardiac metabolism, contractility, ion channel expression, and stress responses (Durgan and Young, 2010; Fishbein et al., 2021; Kelters et al., 2025; Ruan et al., 2021). Experimental disruption of the cardiomyocyte circadian clock dysregulates the expression of *Scn5a* (encoding the voltage-gated sodium channel NaV1.5) and *Kcnh2* [encoding the human ether-à-go-go-related gene (hERG) potassium channel] and increases susceptibility to arrhythmia (Schroder et al., 2013; Schroder et al., 2015). The onset and symptomatic activity of human AF also exhibit diurnal variation, and reduced rest–activity rhythm amplitude has been significantly associated with incident AF (Chen et al., 2014; Yang et al., 2023a). Nevertheless, it remains unclear which circadian-associated transcripts are dysregulated in the diseased human atria, the reproducibility of these expression alterations across independent clinical cohorts, and their specific cellular origins.

Angiopoietin-like 2 (ANGPTL2) and ceruloplasmin (CP) link this temporal research question to two well-characterized dimensions of atrial remodeling. ANGPTL2 is a secreted inflammatory and metabolic regulator that promotes endothelial dysfunction and adverse cardiac remodeling (Tabata et al., 2009; Horio et al., 2014; Tian et al., 2016). CP is a copper-transporting ferroxidase and acute-phase protein that connects metal homeostasis, redox balance, and inflammation (Liu et al., 2022; Yang et al., 2023a). These functions render both genes biologically plausible candidates for involvement in AF; however, biological plausibility alone is insufficient to confirm that they capture reproducible disease-associated remodeling across independent atrial tissue samples. Accordingly, an effective gene prioritization strategy must integrate external replication with temporal and cellular evidence while maintaining a clear distinction between statistical association, biological interpretation, and causal mechanism.

Here, we implemented an evidence-stratified workflow to identify circadian rhythm-associated AF candidate genes and test whether the prioritized association signals were replicable beyond the discovery dataset. Differential expression analysis, circadian-associated gene intersection, co-expression analysis, and convergent machine learning-based feature selection were performed on the GSE115574 dataset. Next, candidate gene expression and discriminative power for AF were evaluated in GSE41177; temporal expression support was examined in heart atrial appendage tissues from the Genotype-Tissue Expression (GTEx) project; cellular distribution was assessed in single-nucleus RNA sequencing data from GSE238242; and transcript and protein abundance were quantified in independent human atrial tissue samples and a cAMP response element modulator (CREM)-mediated atrial arrhythmia mouse model. We additionally performed a screen of a cross-cohort AF expression signature to identify compounds predicted to reverse the disease-associated transcriptional state. When no compound satisfied the prespecified multi-source advancement criteria, we retained the negative finding and separately evaluated whether clinically used antiarrhythmic drugs exhibited predicted structural compatibility with ANGPTL2 or CP. Our study design separated candidate discovery, drug-reversal screening, and hypothesis-driven molecular docking, rather than integrating these steps into a single validation pipeline.

## Materials and methods

### Study design and hierarchy of evidence

The study was designed to stratify analyses into distinct sequential stages: candidate discovery, external replication, temporal pattern validation, cellular localization analysis, and experimental expression verification. This study constitutes an exploratory, evidence-stratified investigation of circadian-associated gene signals in AF. The analytical framework is divided into five core modules: candidate discovery, external replication, orthogonal assessment of tissue-level rhythmicity, cellular localization analysis, and experimental expression verification. No prospective sample size calculation was conducted for the small-scale molecular validation cohort. Dataset GSE115574 was exclusively used for the candidate discovery stage, while GSE41177 was reserved for external transcriptomic validation (Supplementary Figure S2).

### Transcriptomic discovery

To characterize the initial AF-associated atrial transcriptomic landscape, GSE115574 was used exclusively as the discovery cohort. GSE115574 was downloaded from the National Center for Biotechnology Information Gene Expression Omnibus (GEO) (Edgar, 2002). The original source study profiled left atrial and right atrial tissues from patients with severe primary mitral regurgitation, who were stratified into permanent AF and sinus rhythm (SR) groups, using the Affymetrix Human Genome U133 Plus 2.0 array. The discovery dataset in this study comprised 29 samples in total, among which 15 are SR control samples and 14 are AF samples. Expression data were normalized using the robust multi-array average algorithm (Irizarry et al., 2003). Differential expression analysis was performed using the limma package (Smyth, 2004; Ritchie et al., 2015) with an AF-versus-control contrast. We used the following criteria to define exploratory differentially expressed genes (DEGs): nominal two-sided P < 0.05 and absolute log_2_ fold change ≥0.263, which corresponds to an approximately 1.2-fold change in expression magnitude. Benjamini–Hochberg adjusted false discovery rate (FDR) values were calculated separately and reported as a supplementary sensitivity analysis, rather than being used to replace the prespecified discovery criteria.

### Circadian rhythm-associated gene set and enrichment analysis

To link the AF-associated expression signature to temporal biological processes, the DEGs identified in the discovery cohort were intersected with a comprehensive list of circadian-related candidate genes, and the resulting gene set was assessed for functional coherence. The comprehensive candidate gene list was generated by searching GeneCards using the keyword “circadian rhythm,” followed by deduplication to retain 1,732 unique official human gene symbols (Stelzer et al., 2016). This list was applied as a hypothesis-generating resource rather than evidence that every gene on the list is rhythmically transcribed in the human atrium. We intersected the discovery cohort DEGs with this candidate list. Gene Ontology (GO) and Kyoto Encyclopedia of Genes and Genomes (KEGG) pathway enrichment analyses were performed using the clusterProfiler package (Wu et al., 2021) and the org.Hs.eg.db annotation database, with the Benjamini–Hochberg method for multiple testing correction. Protein–protein interaction (PPI) networks were constructed using the Search Tool for the Retrieval of Interacting Genes/Proteins (STRING) database (Szklarczyk et al., 2025) and visualized using Cytoscape (Shannon et al., 2003).

### Weighted gene co-expression network analysis

To identify whether circadian-associated signaling signatures are embedded in coordinated atrial fibrillation-related co-expression modules, weighted gene co-expression network analysis (WGCNA) was performed. WGCNA was performed on the highest-variance quartile of genes following quality filtering using the goodSamplesGenes algorithm (Langfelder and Horvath, 2008). Candidate soft-thresholding powers ranging from 1 to 20 were assessed using the pickSoftThreshold function. A power value of 9 was selected because it satisfied the prespecified scale-free topology fit cutoff of approximately 0.80 while maintaining sufficient network connectivity. Signed adjacency matrices and topological overlap matrices were constructed, followed by average-linkage hierarchical clustering. Dynamic tree cutting was implemented with the parameters deepSplit = 2, pamRespectsDendro = FALSE, and a minimum module size of 50. Modules with eigengene dissimilarity ≤0.25 were merged. Module eigengenes were correlated with AF status via Spearman rank correlation. The AF-associated modules were intersected with the set of circadian rhythm-related DEGs.

### Feature selection and discovery cohort stratification

To prioritize candidate genes retained under multiple distinct statistical assumptions, three complementary feature-selection algorithms were applied to the identified intersecting genes. The 13 intersecting genes were evaluated using least absolute shrinkage and selection operator (LASSO) logistic regression (Tibshirani, 1996), ten-fold cross-validated support vector machine-recursive feature elimination (SVM-RFE; Guyon et al., 2002), and a 1000-tree random forest algorithm (Breiman, 2001). Random forest classification was conducted using the randomForest package in R. A two-level categorical response variable—sinus rhythm (SR) versus atrial fibrillation (AF)—was modeled, and a classification forest comprising 1,000 decision trees was trained with default splitting criteria and bootstrap resampling. Variable importance was quantified using the Mean Decrease Gini index, and predictor genes were ranked accordingly. To ensure full reproducibility, a fixed random seed [set.seed(2025)] was applied prior to model training. Genes retained using all three algorithms were defined as prioritized candidate genes. Single-gene receiver operating characteristic (ROC) curves and a two-gene logistic regression model were fitted in the GSE115574 dataset using the R package pROC (Robin et al., 2011). Classification performance was quantified using the area under the ROC curve (AUC). Of note, the AUC reported here is an apparent in-cohort estimate derived from the discovery cohort and thus cannot be interpreted as valid prospective diagnostic performance.

### Independent external transcriptomic validation

To verify the reproducibility of the two prioritized signals in an independent cohort outside the selection dataset, we evaluated ANGPTL2 and CP in the GSE41177 dataset without performing additional gene selection. The GSE41177 dataset was retrieved from GEO and was generated using the Affymetrix Human Genome U133 Plus 2.0 platform (GPL570). The dataset contains 38 atrial tissue microarrays: 6 from control tissue samples and 32 from persistent AF tissue samples. All samples were derived from the paired left atrial appendage and left atrial–pulmonary vein junction specimens collected from 3 control participants and 16 AF patients. Thus, the primary unit of analysis for the exact replication analysis was the tissue microarray, rather than the individual participant. The robust multi-array average expression matrix provided by GEO was processed with between-array quantile normalization using the limma package.

For each candidate gene, the difference in expression between AF and control samples was summarized as the mean log_2_ expression difference and the corresponding fold change. Two-sided Welch’s t-tests were used to generate primary unadjusted P-values, and Benjamini–Hochberg FDR corrections were performed across the two target genes to obtain supplementary multiplicity adjustment information. External replication was defined *a priori* as meeting the criteria of an absolute log_2_ fold change of at least 0.263 and an unadjusted P < 0.05, which is consistent with the discovery-stage screening rule. The discriminatory power of the single-gene signature was quantified via ROC analysis, with effect sizes reported as the AUC with corresponding 95% confidence intervals.

### GTEx atrial rhythmicity

To obtain temporal expression support independent of the AF case–control dataset, we examined candidate rhythmicity in the GTEx heart atrial appendage resource. Specifically, tissue-level circadian evidence was retrieved from the rhythmic expression dataset of atrial appendage tissues provided by GTEx. ANGPTL2 and CP were classified based on the reported rhythmicity q-values and phase estimates from the existing literature (Ruben et al., 2018). This analysis was designed to detect whether the target genes exhibit a rhythmic expression signal in non-AF human atrial tissues, and it does not address AF-specific rhythmicity, circadian phase shifts, or causal regulation by the circadian clock.

### Immune deconvolution and candidate-defined molecular states

To explore the biological states associated with the expression of candidate genes, we performed immune deconvolution, pathway scoring, and candidate-based clustering as hypothesis-generating analyses. Specifically, Cell-type Identification by Estimating Relative Subsets of RNA Transcripts (CIBERSORT) with the LM22 reference matrix was applied to estimate the proportions of 22 immune cell subsets in the discovery expression matrix (Newman et al., 2015). This analysis was conducted with 100 permutations, quantile normalization, and a fixed random seed. Cell types with zero estimated abundance across all samples were excluded from downstream analysis. Group comparisons were performed using two-tailed statistical tests, and Spearman correlation analysis was conducted to assess the associations between candidate gene expression and inferred immune cell fractions in AF samples, with multiple testing corrected using the Benjamini–Hochberg procedure.

Gene set variation analysis (GSVA) was performed on AF samples using Hallmark gene sets retrieved from the Molecular Signatures Database (MSigDB) v2024.1 (Hänzelmann et al., 2013). The samples were stratified into high-expression and low-expression groups based on the median expression level of each candidate gene. ConsensusClusterPlus analysis was conducted on the 14 AF samples using the expression profiles of ANGPTL2 and CP (Wilkerson and Hayes, 2010). Principal-component analysis (PCA) and gene set enrichment analysis (GSEA) were subsequently performed to characterize the two-cluster classification (Subramanian et al., 2005).

### Single-nucleus localization

To identify the atrial cell populations that contribute to the bulk candidate signal, we localized the expression of *ANGPTL2* and *CP* in a left atrial appendage single-nucleus RNA sequencing (snRNA-seq) dataset. The dataset contains paired single-nucleus RNA and chromatin profiling data from left atrial appendage samples from 4 donors with sinus rhythm and three donors with persistent AF. Only the snRNA-seq modality was analyzed in the present study. After filtering out 57 low-quality nuclei, the retained Seurat object contained 11,929 high-quality nuclei. Raw read counts were normalized to 10,000 transcripts per nucleus and transformed using the log1p approach. For uncorrected cell localization analysis, we used 3,000 highly variable genes, 50 principal components, a k-nearest neighbor graph constructed with k = 15, and a Leiden clustering resolution of 0.6 (Leblanc et al., 2024; Hao et al., 2021). Cell type annotation was performed based on canonical cell type markers and one-versus-rest Wilcoxon rank sum tests. The present analysis performed descriptive localization of candidate genes at the donor level; nuclei were not treated as independent biological replicates, and no donor-aware case–control differential expression analysis was conducted.

### Human left atrial appendage validation cohort

To validate the expression of candidate genes in an independent clinical cohort, left atrial appendage tissue was collected from patients with persistent AF and individuals with sinus rhythm. All left atrial appendage specimens were collected at Nanjing Drum Tower Hospital between September 2021 and May 2022 from patients undergoing open-heart surgery. The AF group consisted of five patients diagnosed with persistent AF, defined as AF lasting for at least 7 days (Joglar et al., 2024), with preserved left ventricular ejection fraction (LVEF). The SR control group consisted of five age-matched patients undergoing valve surgery who presented with SR preoperatively. The eligibility criteria required available preoperative electrocardiography (ECG), Holter monitoring, echocardiography results, and complete clinical history. Participants with LVEF < 50% or E/e′ >15 were excluded from the analysis. SR controls were required to remain free of new-onset AF throughout the postoperative follow-up period. Comorbidities of all participants are summarized in Table 1.

**TABLE 1:** Baseline characteristics of the human atrial-tissue validation cohort.

Characteristic	SR (n = 5)	AF (n = 5)	P value
Age, years	66 ± 6	69 ± 6	0.527
Male sex, n (%)	4 (80%)	1 (20%)	0.206
BMI, kg/m^2^	24.8 ± 2.4	23.8 ± 3.5	0.593
Hypertension, n (%)	3 (60%)	2 (40%)	1.000
Diabetes, n (%)	1 (20%)	0 (0%)	1.000
Coronary artery disease, n (%)	1 (20%)	0 (0%)	1.000
Smoking, n (%)	0 (0%)	1 (20%)	1.000
Alcohol use, n (%)	0 (0%)	0 (0%)	1.000
LAD, cm	4.25 ± 0.47	5.87 ± 0.90	0.012
LVEF, %	56.9 ± 5.1	57.8 ± 4.3	0.771
Blood transfusion, mL	400 (50–1775)	50 (50–550)	0.504
Operation time, min	270 (260–320)	295 (270–315)	0.834
Aortic cross-clamp time, min	148 (133–160)	137 (125–140)	0.531
CPB time, min	180 (179–210)	160 (158–164)	0.296
NYHA class, n (%)	​	​	0.444
I	0 (0%)	0 (0%)	​
II	2 (40%)	0 (0%)	​
III	3 (60%)	5 (100%)	​
IV	0 (0%)	0 (0%)	​
CRP, mg/L	4.1 (1.7–13.9)	3.9 (1.6–4.7)	0.463
Hemoglobin, g/L	127.0 (115.0–142.0)	125.0 (123.0–134.0)	1.000
ALT, U/L	16.9 (10.3–22.6)	19.2 (14.4–20.1)	1.000
TBA, µmol/L	7.0 (4.2–7.2)	9.0 (3.0–10.3)	0.917
Serum creatinine, µmol/L	75.0 (69.0–87.0)	80.0 (57.0–87.0)	0.917
BUN, mmol/L	5.9 (5.4–7.7)	7.1 (6.5–7.7)	0.463
BNP, pg/mL	307.0 (43.1–443.0)	179.0 (169.0–237.0)	1.000

Data are represented as mean ± SD, median (interquartile range), or n (%). Continuous variables were compared using two-sided Welch tests or Mann–Whitney U tests, as appropriate; categorical variables were compared using Fisher exact tests. Baseline P-values are unadjusted and descriptive. AF, atrial fibrillation; ALT, alanine aminotransferase; BMI, body mass index; BNP, B-type natriuretic peptide; BUN, blood urea nitrogen; CPB, cardiopulmonary bypass; CRP, C-reactive protein; LAD, left atrial diameter; LVEF, left ventricular ejection fraction; NYHA, New York Heart Association; SD, standard deviation; SR, sinus rhythm; TBA, total bile acids.

AF was identified by the absence of P waves and irregular R–R intervals on 12-lead ECG. Excised tissue specimens obtained during surgery were snap-frozen in liquid nitrogen immediately after resection and stored at −80 °C for subsequent analysis. This study was approved by the Institutional Review Board of Nanjing Drum Tower Hospital (approval number: 2020-185-01), and written informed consent was obtained from all participants prior to enrollment.

### CREM mouse validation

To investigate whether the observed expression pattern is conserved in an atrial arrhythmia model, we measured the levels of candidate transcripts and proteins in the atria of CREM mutant mice and wild-type controls. Atrial tissues were collected from 28-week-old male wild-type mice and male heterozygous cardiac-specific alpha-myosin heavy chain (α-MHC)-CREM-IbΔC-X-PolyA Cas9 knock-in mice (n = 6 per group), a well-characterized model of spontaneous AF (Chen et al., 2024). Genotyping was performed using three polymerase chain reaction (PCR) assays targeting the 5′ arm, 3′ arm, and wild-type locus (Supplementary Figure S1A). Surface ECG recordings were acquired from anesthetized mice maintained on isoflurane (Supplementary Figures S1B,C). All animal experiments were approved by the Experimental Animal Welfare Ethics Committee of Nanjing Drum Tower Hospital (approval number: 2026AE010462; approval date: 6 July 2026).

### Quantitative PCR, immunoblotting, and statistics

To validate candidate abundance across multiple molecular layers, targeted transcript and protein measurements were analyzed with biological replicates set as the statistical units. Total RNA was isolated using the TRIzol reagent, reverse-transcribed, and quantified using SYBR Green-based real-time quantitative PCR (qPCR; Bustin et al., 2009). Cycle threshold values from technical qPCR replicates were averaged prior to downstream statistical analysis. Protein abundance was quantified using immunoblotting, normalized to the glyceraldehyde-3-phosphate dehydrogenase (GAPDH) loading control, and further normalized to the mean value of the corresponding control group. To reduce the risk of inflated type I error from pseudo-replication, biological replicates, rather than independent technical measurements, were defined as the primary statistical units. Two-sided Welch’s t-tests were used for prespecified targeted comparisons of gene expression. For genome-wide and multi-cell analyses, the Benjamini–Hochberg procedure was used for multiple testing correction; nominal P-values were retained for exploratory associations that were explicitly labeled as such. No experimental outliers were excluded from the analysis.

### Cross-cohort drug-reversal screening

To identify whether the AF-associated transcriptional signature can prioritize candidate compounds for further investigation, we conducted a constrained cross-cohort drug-reversal analysis prior to molecular docking. The datasets GSE115574, GSE282504, and GSE69890 were analyzed independently, without merging expression matrices or pooling fitted regression coefficients. Within each cohort, genes were filtered using the prespecified threshold: P < 0.05 and absolute log_2_ fold change ≥0.263. We then performed a gene-level random-effects meta-analysis for genes detected in at least two cohorts. Consensus core genes were required to satisfy the following criteria: (1) meet the cohort-level significance threshold with the same direction of expression change across at least two cohorts; (2) no statistically significant expression change in the opposite direction at any cohort-level threshold; (3) a meta-analysis P < 0.05. Finally, the top 50 upregulated and top 50 downregulated consensus genes were compiled to form the AF disease signature.

The top-50 gene signature was submitted to the L1000 fireworks display (L1000FWD) platform to identify drug-induced transcriptional profiles that exhibit opposite expression patterns to the AF signature (Lamb et al., 2006; Subramanian et al., 2017; Wang et al., 2018). The retrieved named compounds were further cross-validated against manually curated drug signatures from the Crowd Extracted Expression of Differential Signatures (CREEDS) database and target-perturbation records from the integrative LINCS (iLINCS) database (Wang et al., 2016; Pilarczyk et al., 2022). To qualify as a candidate compound, a molecule was required to meet three preset criteria: reaching the statistical significance threshold for FDR in L1000FWD analysis, showing independent directional reversal of the AF signature in CREEDS validation, and having available interpretable target evidence or translational research support. The candidate screening workflow terminated once no remaining compounds satisfied all of the above criteria, and consequently, no compounds derived from this *in silico* screening were carried forward to molecular docking.

### Exploratory molecular docking of clinically used antiarrhythmic drugs

After the primary screening yielded no compounds suitable for further development, we pursued a separate hypothesis-driven question: whether clinically approved antiarrhythmic drugs can occupy the predicted binding pockets of ANGPTL2 or CP under a simplified structural model. Amiodarone, flecainide, and propafenone were selected *a priori* because they are clinically used for pharmacological rhythm control in AF (Joglar et al., 2024). This selection was independent of the initial drug-reversal screen, and these results are not presented as a drug repurposing outcome. Molecular docking was performed as an exploratory analysis of structural compatibility and was not part of the primary validation workflow. Receptor structures, ligand preparation, search grid parameters, AutoDock Vina settings, and predicted binding poses are provided in Supplementary Methods and Supplementary Figure S3 (Trott and Olson, 2010; Ferreira et al., 2015). Docking scores below −5.0 kcal mol^−1^ were set as a pragmatic threshold for pose ranking and were not interpreted as evidence of experimental binding, target engagement, target expression reversal, or clinical efficacy against AF.

## Results

### The discovery cohort captured a broad atrial transcriptomic program associated with AF

To characterize the initial AF-associated transcriptomic expression landscape, we first analyzed the GSE115574 discovery cohort, which included 15 SR control samples and 14 AF patient samples. Using the prespecified nominal P-value and effect-size threshold, we identified 479 differentially expressed transcripts in the AF versus control contrast, of which 235 were upregulated and 244 were downregulated (Figure 1A). These transcripts constituted the initial disease-associated candidate space for subsequent biological and network-based refinement.

**FIGURE 1:**
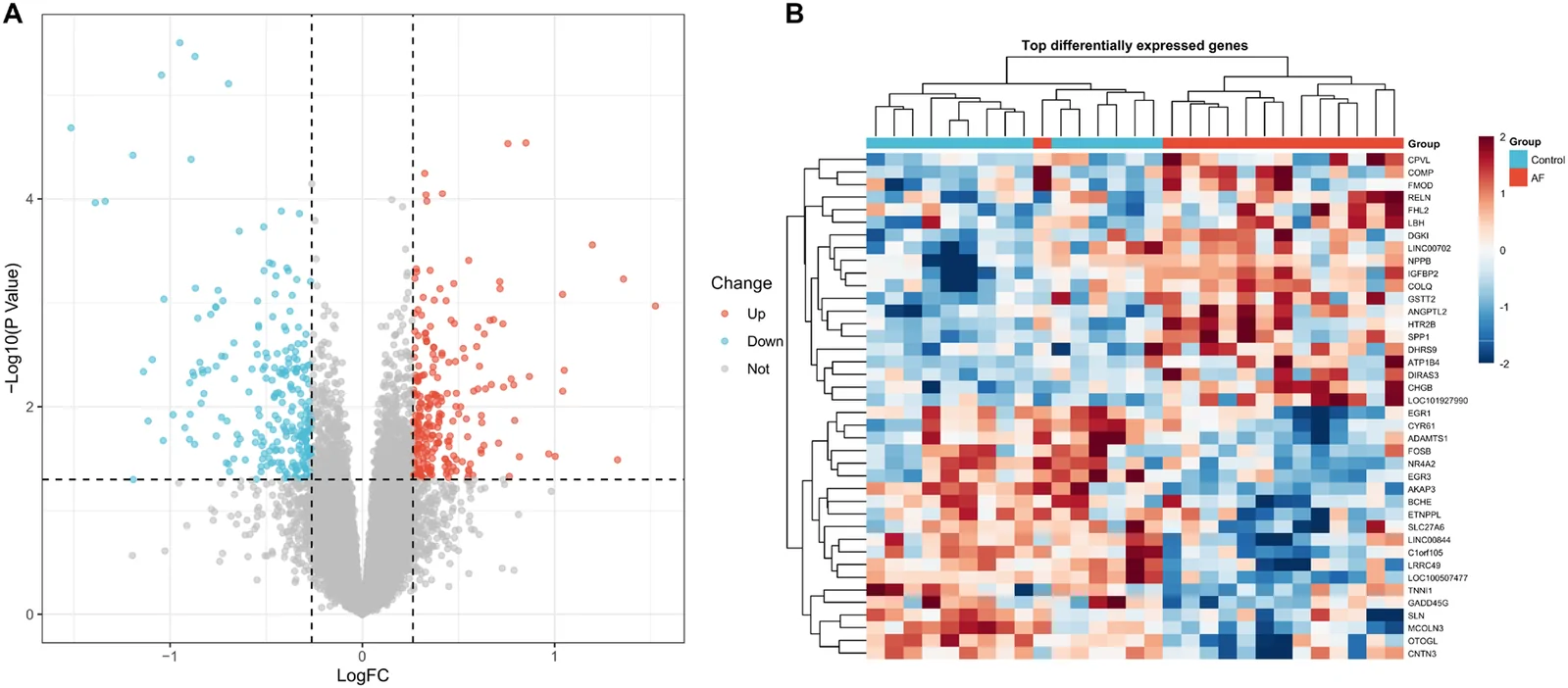
Differential expression analysis in GSE115574. **(A)** Volcano plot of the AF-versus-control comparison. **(B)** Heatmap of the leading differentially expressed genes in AF and control atrial samples.

The top differentially expressed genes successfully separated the majority of AF and SR samples while retaining detectable intragroup heterogeneity (Figure 1B). This pattern—intergroup separation accompanied by intragroup heterogeneity—supports the view of AF as a remodeling syndrome shaped by multiple, distinct atrial pathophysiological processes, rather than a uniform transcriptional state.

### Circadian-associated transcripts were enriched for rhythmicity, inflammation, and stress response

To associate the disease-specific expression signature with temporal biological processes, we overlapped the 479 discovery-phase transcripts with 1,732 circadian-related genes curated from GeneCards. This filtering step narrowed the candidate list to 55 genes that connect AF-associated differential expression with experimentally validated circadian relevance (Figure 2A). PPI analysis revealed that most of the 55 candidates were embedded within a single connected functional network, demonstrating that the retained genes share a common biological context rather than constituting a set of independent isolated markers (Figure 2B).

**FIGURE 2:**
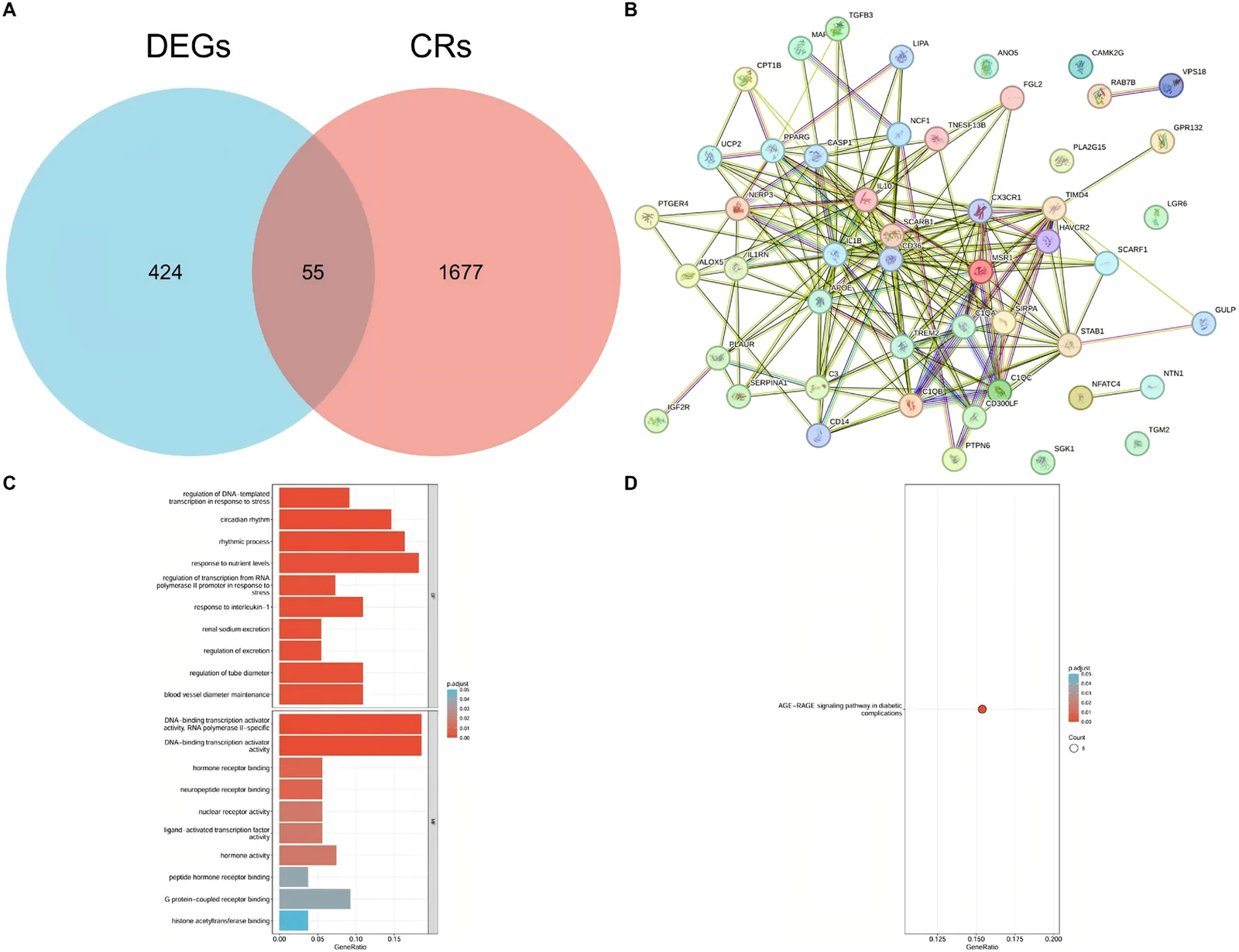
Identification and functional annotation of circadian-associated AF genes. **(A)** Intersection of AF-related differentially expressed genes and circadian-associated genes. **(B)** PPI network of the intersecting genes. **(C)** GO enrichment analysis. **(D)** KEGG pathway enrichment analysis.

GO enrichment analysis associated the candidate genes with rhythmic processes, cytokine-mediated signaling responses, stress-activated transcriptional regulation, and receptor-binding functions (Figure 2C). KEGG pathway analysis identified advanced glycation end product-receptor for advanced glycation end products (AGE-RAGE) signaling as a key enriched pathway among the retained candidates (Figure 2D). Collectively, these findings position temporal regulation as a core feature co-occurring with inflammatory and metabolic stress responses in the atrial fibrillation-associated transcriptomic program. The convergence of these functional categories indicates that the circadian-related component of the atrial fibrillation-associated transcriptomic signal is not restricted to canonical clock genes; rather, temporal regulatory organization may be integrated into the inflammatory and metabolic responses that drive atrial remodeling.

### Co-expression analysis enriched circadian-associated signals in an AF-linked module

To determine whether circadian-associated signals are embedded in coordinated AF-related modules, we first confirmed that sample clustering did not identify any outlying samples requiring removal prior to network construction (Figure 3A). Evaluation of candidate soft-thresholding powers revealed that a power of 9 achieved the prespecified target for approximate scale-free topology while retaining adequate mean connectivity (Figure 3B). Dynamic tree cutting and module merging yielded the final set of signed co-expression modules (Figure 3C). The magenta module exhibited the strongest positive association with AF (Figure 3D), concentrating disease-associated signal within a coordinated expression neighborhood.

**FIGURE 3:**
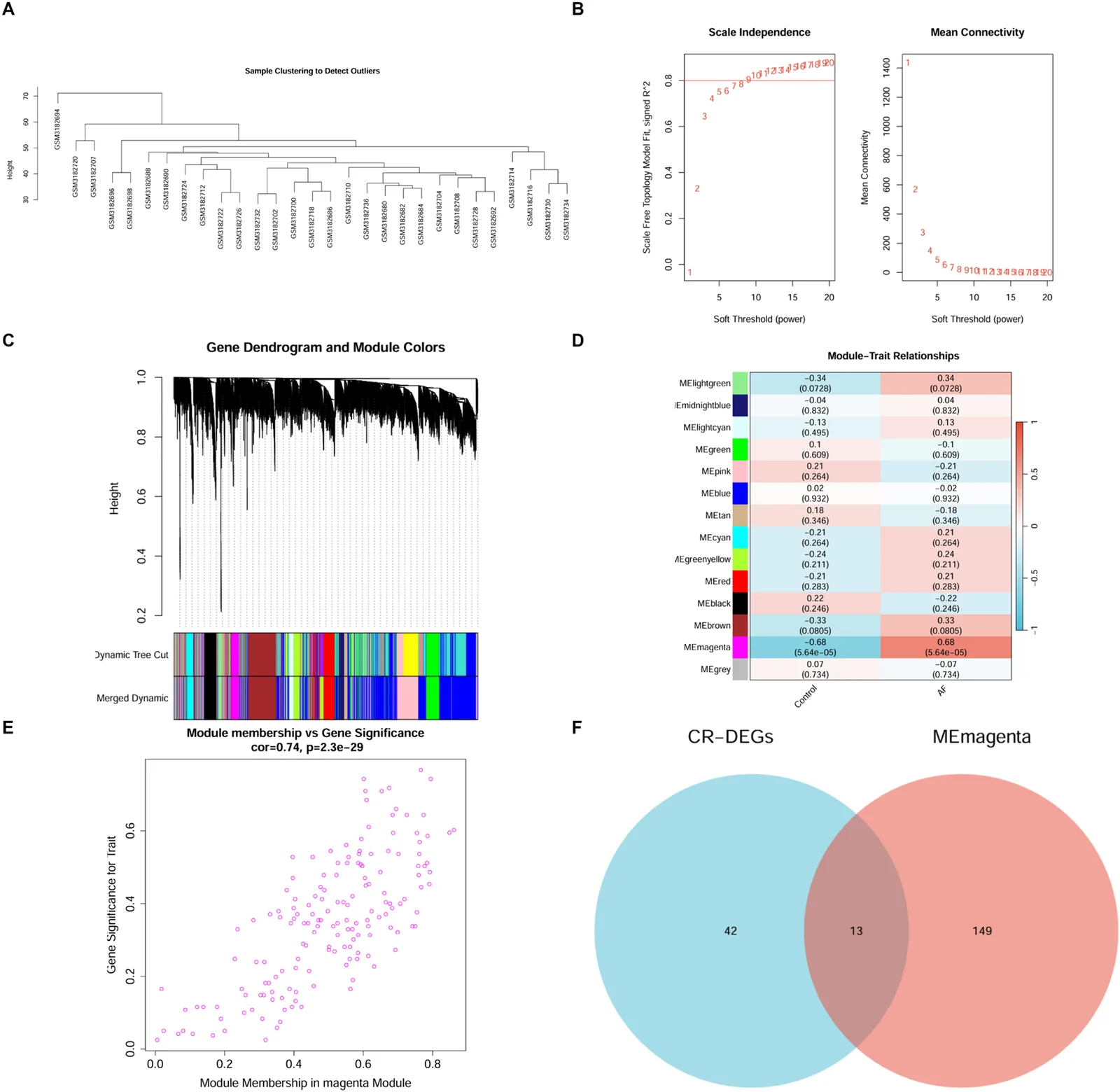
WGCNA of circadian-associated AF genes. **(A)** Sample-clustering dendrogram. **(B)** Scale-free topology fit and mean connectivity for soft-threshold selection. **(C)** Gene dendrogram and module assignment. **(D)** Module–trait relationships. **(E)** Correlation between module membership and gene significance in the magenta module. **(F)** Intersection of circadian-associated differentially expressed genes and magenta-module genes.

Within the magenta module, module membership significantly correlated with gene significance (Figure 3E), suggesting that hub genes—those with higher intramodular connectivity—exhibit stronger associations with AF. Overlap between the 162 magenta-module genes and the 55 circadian-associated DEGs yielded a refined set of 13 candidate genes (Figure 3F).

### Three complementary feature-selection strategies converged on ANGPTL2 and CP

To prioritize genes that were robust to model selection, we implemented three complementary algorithms. LASSO retained six genes after penalized model fitting and cross-validation (Figures 4A,B). This analysis favors a sparse set of predictors under a linear logistic framework. SVM-RFE obtained its optimal feature set consisting of two genes when jointly considering cross-validated prediction error and classification accuracy (Figures 4C,D). In the revised 1,000-tree classification random forest, AF/SR status was modeled as a categorical factor, and all 13 input genes exceeded the prespecified MeanDecreaseGini >0.5 threshold (Figure 4E). ANGPTL2 ranked first and CP ranked third according to MeanDecreaseGini.

**FIGURE 4:**
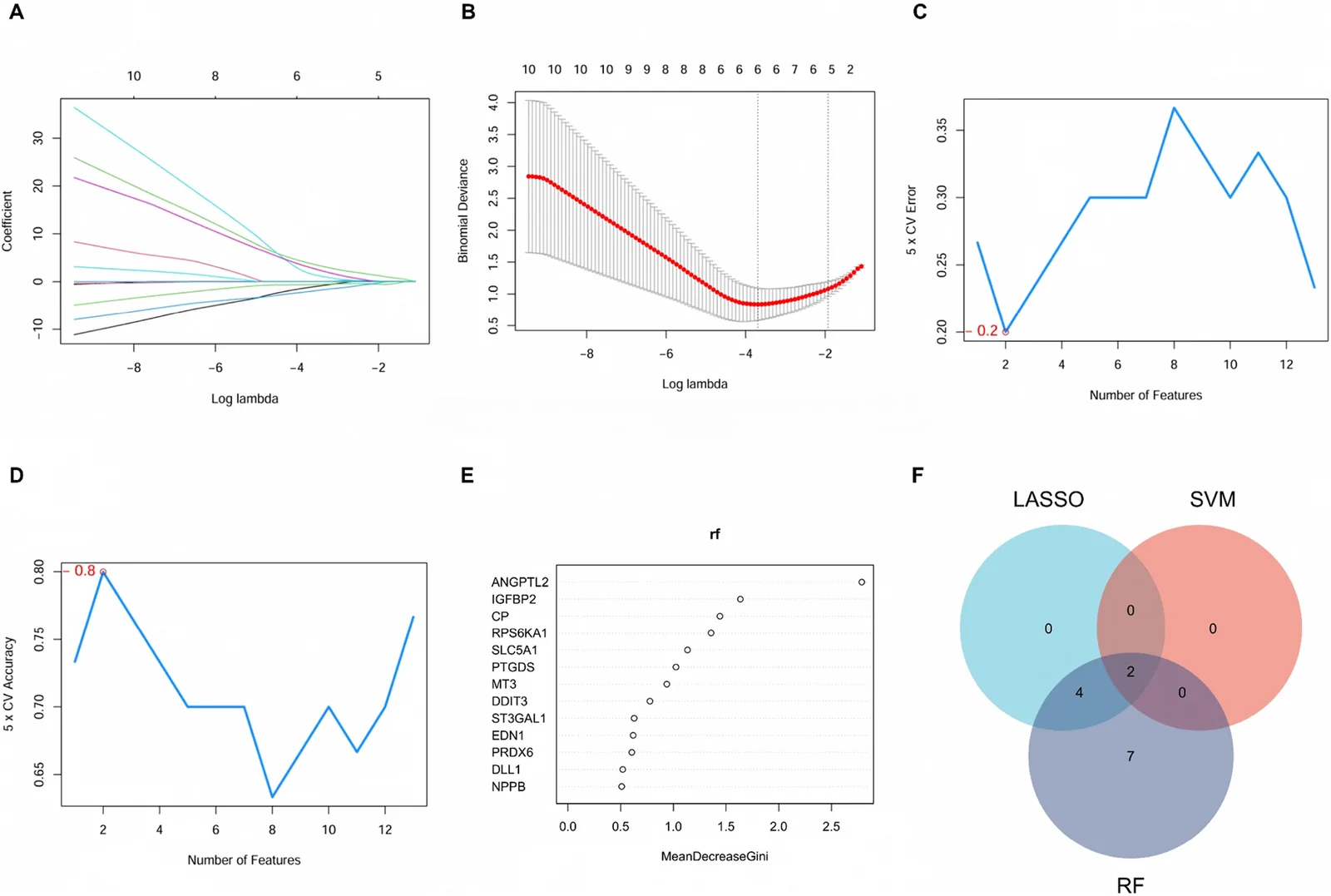
Machine-learning selection of candidate genes. **(A)** LASSO coefficient profiles. **(B)** Ten-fold cross-validation for LASSO variable selection. **(C,D)** Cross-validated error and accuracy for SVM-RFE feature selection. **(E)** Variable importance from the 1,000-tree classification random forest, ranked by MeanDecreaseGini. **(F)** Intersection of genes selected by LASSO, SVM-RFE, and the classification random forest.

ANGPTL2 and CP were the only two genes concurrently identified using LASSO, SVM-RFE, and the classification random forest (Figure 4F). The concordance of this result across three algorithms based on distinct statistical assumptions, rather than the variable ranking generated using any single method, formed the rationale for prioritizing these two genes. From a biological perspective, this gene pair links circadian-related candidate regions to two distinct functional remodeling pathways: ANGPTL2 is associated with inflammatory and vascular-stromal signaling, while CP is involved in copper homeostasis and oxidative-redox regulation. Therefore, the two-gene signature not only retains superior statistical discriminative power but also reveals distinct biological processes through which circadian organization interacts with atrial fibrillation substrate.

### ANGPTL2 and CP were increased in the discovery atria

To evaluate whether the prioritized candidate genes can distinguish AF from SR in the discovery cohort, we compared their expression levels and discriminatory power. The expression of ANGPTL2 and CP was significantly higher in AF samples than in SR samples (Figure 5A). We adopted a prespecified threshold of |log_2_ fold-change| ≥ 0.263, which corresponds approximately to a 1.2-fold change, to retain moderately differentiated signals for downstream integration analysis. Threshold analysis identified 479, 129, and 19 nominally significant genes at absolute log_2_ fold-change cutoffs of 0.263, 0.5, and 1.0, respectively. ANGPTL2 exhibited the strongest effect size across all tested thresholds, so it was prioritized over CP for subsequent external validation.

**FIGURE 5:**
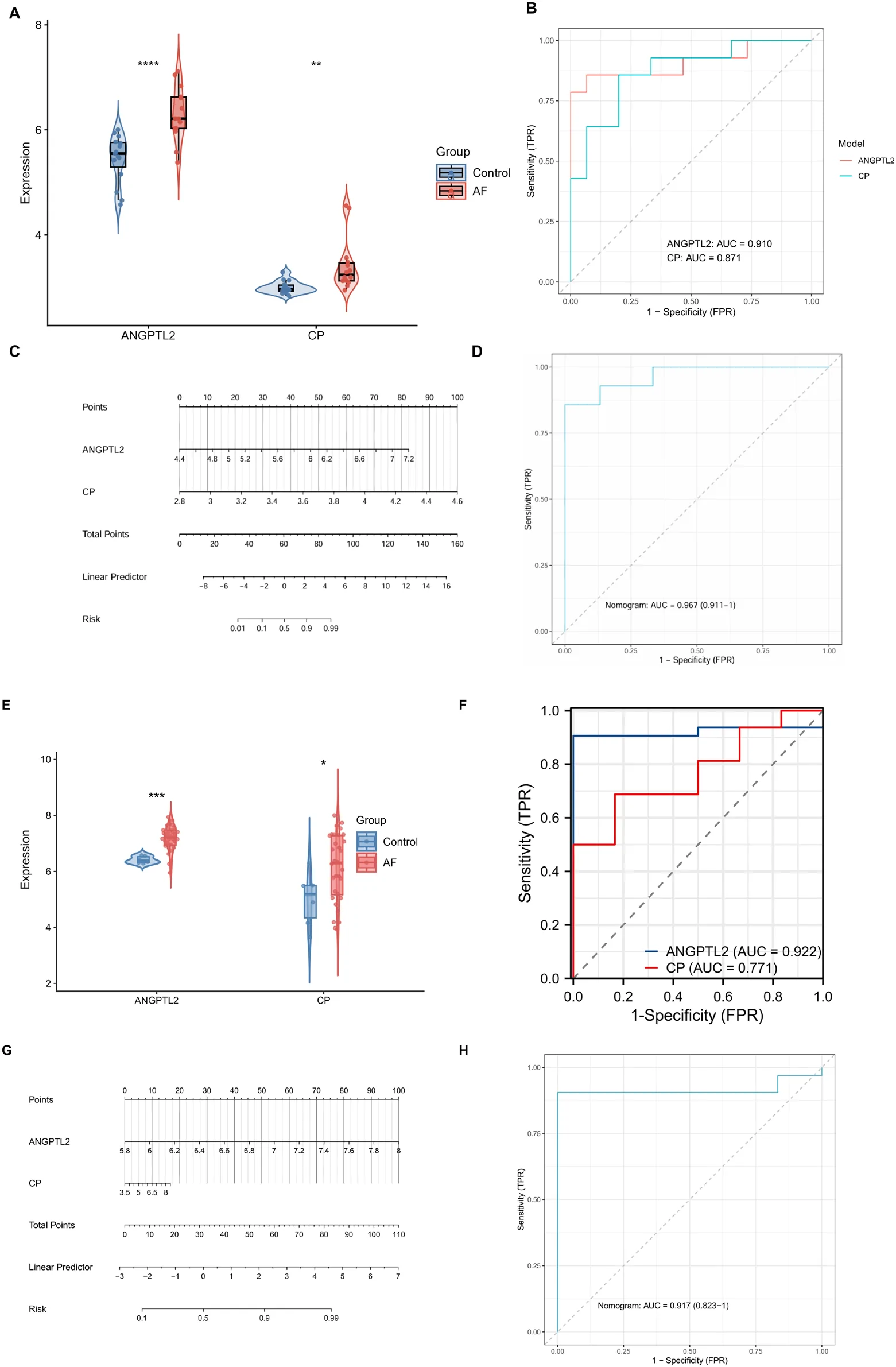
Discovery and GSE41177 validation of ANGPTL2 and CP. **(A)** Discovery-cohort expression distributions of *ANGPTL2* and *CP* in AF and control samples. **(B)** Discovery single-gene ROC curves. **(C)** Discovery two-gene nomogram. **(D)** Apparent discovery ROC curve of the two-gene model. **(E)** ANGPTL2 and CP expression in six control and 32 AF tissue arrays from GSE41177. **(F)** GSE41177 single-gene ROC curves. **(G)** Cohort-specific GSE41177 two-gene nomogram. **(H)** Apparent ROC curve of the cohort-specific GSE41177 two-gene model. The 38 arrays were derived from paired atrial tissues in three control and 16 AF participants and should not be interpreted as 38 independent participants.

ANGPTL2 and CP yielded single-gene AUCs of 0.910 and 0.871, respectively, in the discovery cohort (Figure 5B), indicating that each candidate achieved substantial separation between AF and SR samples in the discovery set. The two-gene regression coefficients were integrated into a nomogram (Figure 5C), and the combined risk score generated an AUC of 0.967 in the discovery cohort (Figure 5D). These discovery-phase estimates established a reference for benchmarking the independent expression validation and discrimination performance obtained in subsequent analyses. Our combined signal further suggests that AF-associated temporal remodeling can be characterized by multiple distinct biological axes, with inflammatory-vascular and copper-redox components contributing complementary prognostic information.

### ANGPTL2 and CP were both reproduced in GSE41177 with distinct performance profiles

To evaluate whether the two discovery signals remained statistically significant in an independent atrial tissue cohort, we analyzed the GSE41177 dataset. Notably, this dataset comprises 38 tissue specimens rather than 38 independent individuals: 6 control tissue samples and 32 persistent AF tissue samples were acquired from paired atrial sites of 3 control individuals and 16 participants with AF. This sampling design enabled an independent tissue-level validation of the two prespecified candidate signals (Figures 5E–H).

ANGPTL2 expression was significantly upregulated in AF tissue, with a log_2_ fold change of 0.760, corresponding to a 1.694-fold increase. This differential expression was statistically supported by a Welch’s P-value of 6.84 × 10^−9^ and a FDR of 1.37 × 10^−8^ (Figure 5E). ANGPTL2 also enabled the discrimination of AF tissue from control tissue with an AUC of 0.922 (Figure 5F). Thus, existing external evidence indicates that ANGPTL2 exhibits both a reproducible expression shift and strong performance in tissue-level classification.

CP was also significantly upregulated in AF tissue and showed a larger expression change than ANGPTL2 in this cohort. The differential expression remained statistically significant after adjustment for multiple testing across the two targets (Figure 5E). CP achieved an AUC of 0.771 (Figure 5F), indicating moderate discriminatory power, in contrast to the near-perfect classification performance of ANGPTL2.

Both candidates exceeded the prespecified absolute log_2_ fold-change threshold of 0.263 and exhibited unadjusted P-values below 0.05. The external validation result thus reproduced both the effect direction and threshold-defined significance of the two discovery signals while resolving their distinct functional profiles: ANGPTL2 conferred stronger discriminatory capacity, whereas CP displayed a larger magnitude of expression shift. This observed complementarity indicates that circadian-associated candidate biomarkers do not necessarily exhibit identical biomarker behavior in remodeled atrial tissue. ANGPTL2 may more consistently distinguish the AF tissue state, while CP may capture a larger yet more heterogeneous copper-redox response across atrial tissue samples. A cohort-specific two-gene nomogram yielded an observed AUC of 0.917 (Figures 5G,H), and this within-cohort fitting result was interpreted as descriptive rather than serving as frozen-model validation.

In the paired-tissue sensitivity analysis, both targets remained upregulated when the participant was modeled as a blocking factor, although different target probes were selected in this adjusted model. ANGPTL2 yielded a log_2_ fold change of 1.397, and CP yielded a log_2_ fold change of 1.416. This sensitivity analysis result is consistent with the effect direction identified in the primary tissue array analysis, without introducing confounding between the two probe design specifications.

### ANGPTL2 and CP showed tissue-level rhythmicity in the human atrial appendage

To obtain temporal support independent of AF case–control expression data, we analyzed the rhythmicity resource derived from GTEx heart atrial appendage samples. ANGPTL2 satisfied the predefined rhythmicity criterion (q = 0.0168743; estimated phase = 6.704 h). CP independently met the prespecified threshold of q < 0.05 (q = 0.0483866; phase = 0.154 h; Supplementary Table S2). These independent temporal data situate both candidate genes within the context of rhythmic transcription in the human atria. Their distinct estimated phases further indicate that ANGPTL2 and CP may reflect distinct temporal components of atrial biology, rather than acting as interchangeable clock-associated markers. From a practical perspective, the measured abundance and observed case–control expression difference of either transcript could be partially dependent on sampling time. Therefore, time-stamped atrial sampling may help distinguish persistent AF-associated elevation from phase- or amplitude-dependent expression variation in future investigations.

### Immune and contractile programs varied across AF samples

To investigate whether the expression of our candidate genes correlates with inflammatory and contractile phenotypic states, we analyzed inferred immune cell proportions and candidate-based cell clusters. We found that estimated immune cell composition exhibited substantial heterogeneity across samples, and nominally significant differences in the proportions of monocytes, memory B cells, M2 macrophages, and resting mast cells were observed between AF and control samples (Figures 6A,D). Within AF samples, the expression levels of ANGPTL2 and CP were nominally correlated with specific inferred immune fractions at P < 0.05 (Figures 6B,C). This association places both candidate genes in an inflammatory cellular microenvironment, which provided the rationale for our subsequent single-cell spatially resolved validation experiments.

**FIGURE 6:**
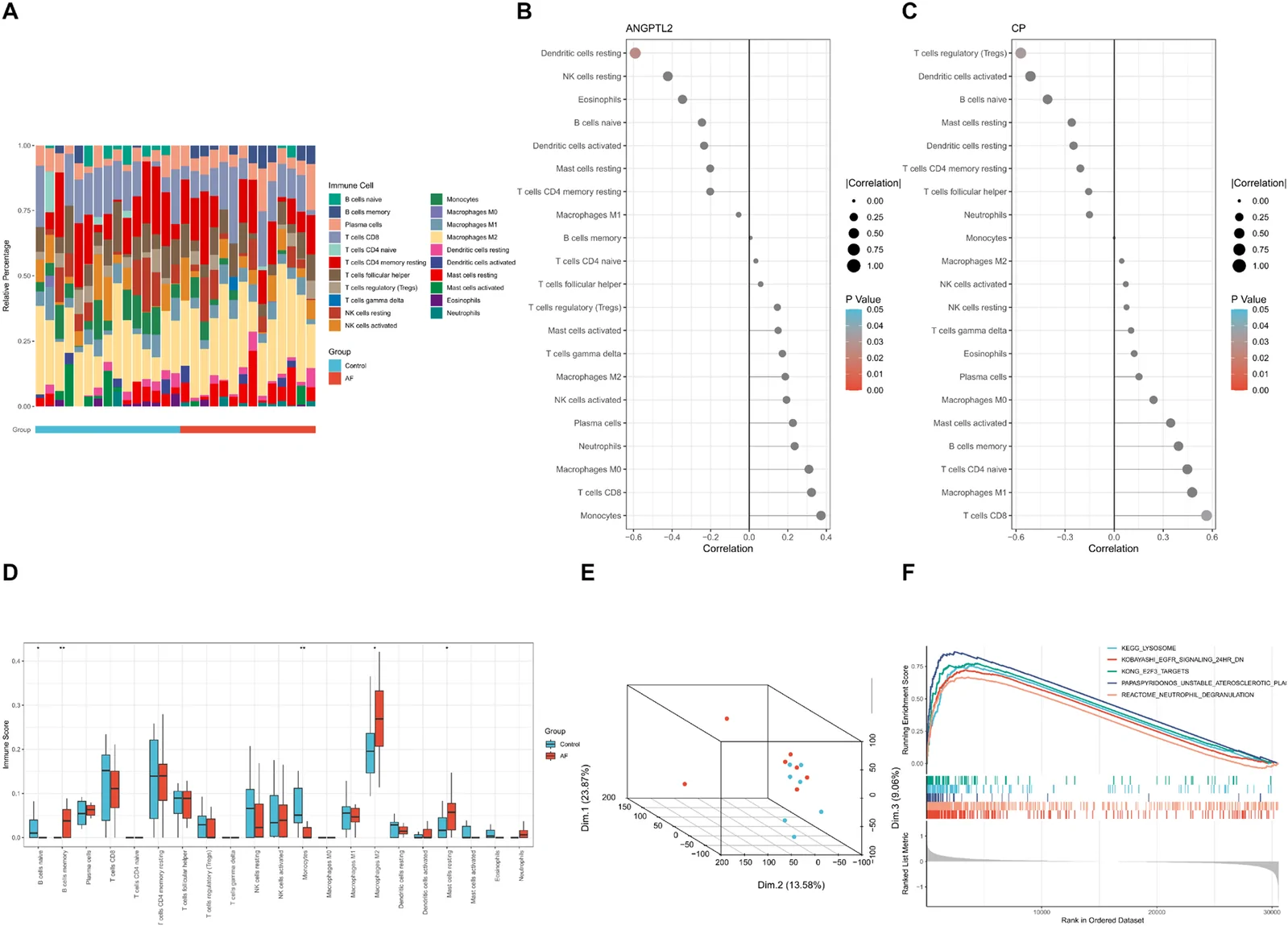
Immune infiltration and candidate-defined molecular states in AF. **(A)** Relative immune-cell fractions across samples. **(B,C)** Correlations of *ANGPTL2* and *CP* with inferred immune cells. **(D)** Comparison of immune-cell fractions between AF and control groups. **(E)** Ordination of the two candidate-defined AF clusters. **(F)** Gene-set enrichment profiles of the clusters.

Consensus clustering based on ANGPTL2 and CP stratified the 14 AF samples into two subgroups of seven samples each (Figure 6E). One subgroup exhibited distinct enrichment profiles related to epidermal growth factor receptor (EGFR) signaling, neutrophil degranulation, and lysosomal pathways, while the other was enriched for programs associated with muscle contraction, vascular smooth muscle contraction, and dilated cardiomyopathy (Figure 6F). These opposing enrichment profiles delineated an exploratory inflammatory–contractile axis within the AF cohort. Since immune activity, vascular tone, and myocardial contractile programs are all temporally organized physiological processes, their stratification by ANGPTL2 and CP establishes a plausible framework, wherein altered temporal regulation is associated with AF heterogeneity, rather than presenting as a uniform feature across all patients.

### Single-nucleus data localized the candidates across vascular and stromal atrial populations

To identify the specific cell populations that express candidate circadian rhythm-associated genes, we analyzed the GSE238242 dataset, which retained 11,929 nuclei from four donors with SR and three donors with persistent AF after quality control (Figures 7A,B). This dataset allowed us to achieve cell-resolved localization of *ANGPTL2* and *CP* in the atrial tissue microenvironment. Low-dimensional embedding and canonical marker expression confirmed the annotation of the major atrial cell populations (Figures 7C,D), and these annotations served as the reference framework for the localization of our candidate genes.

**FIGURE 7:**
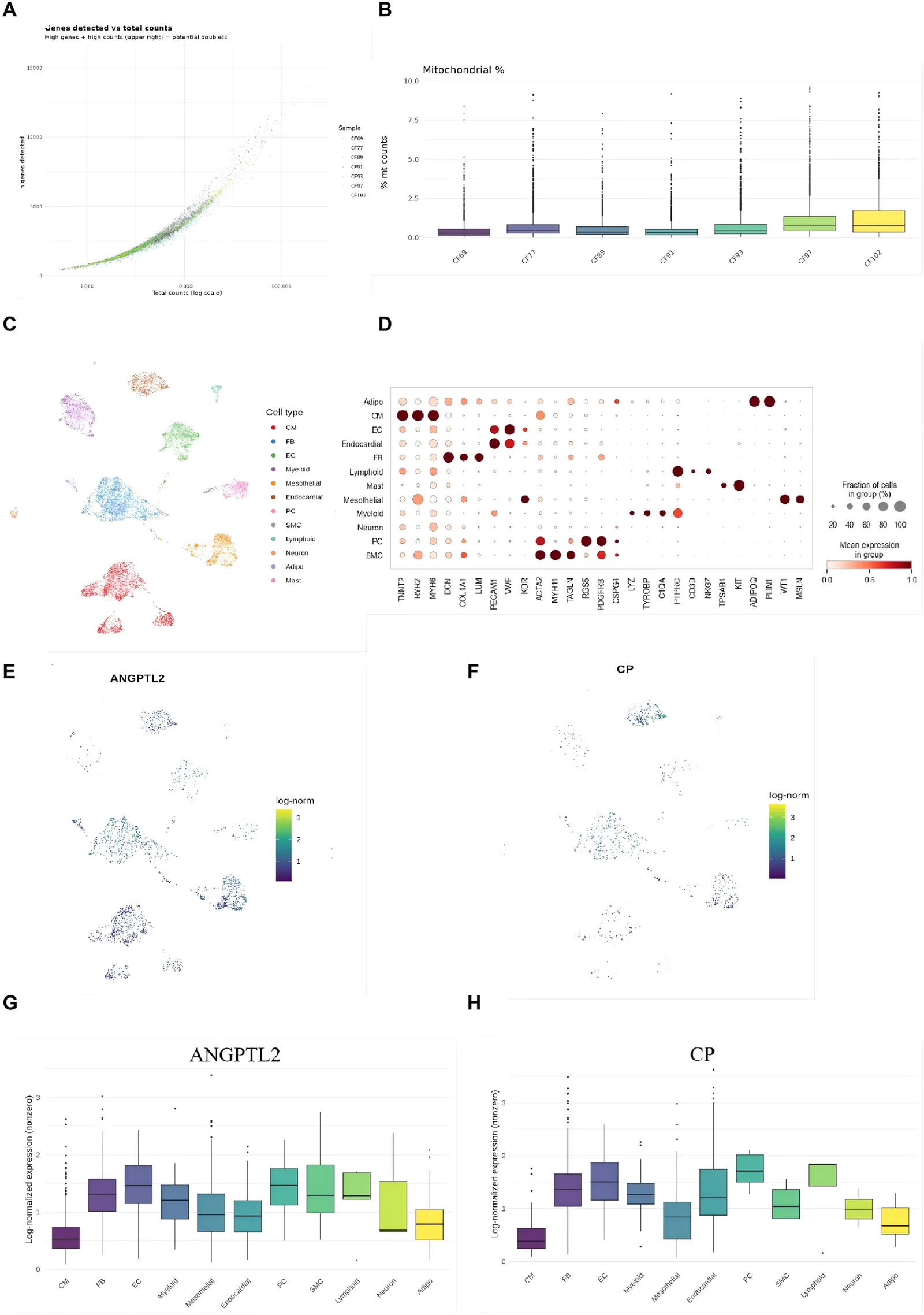
Single-nucleus localization of ANGPTL2 and CP in GSE238242. **(A,B)** Quality-control metrics across nuclei and donors. **(C)** Low-dimensional embedding of annotated atrial cell populations. **(D)** Expression of canonical cell-type markers. **(E,F)** Feature plots of *ANGPTL2* and *CP*. **(G,H)** Expression distributions of *ANGPTL2* and *CP* across cell populations.

ANGPTL2 and CP were detected across multiple noncardiomyocyte populations in both feature plots and cell-type distribution analyses (Figures 7E–H). Relatively high expression signals were observed in vascular and stromal compartments, including pericytes, and subsets of endothelial cells, fibroblasts, and lymphoid cells. This distribution supports a multicellular mechanism interpretation, wherein noncardiomyocyte populations contribute substantially to the bulk ANGPTL2 and CP signals detected in AF atrial tissue. Furthermore, this finding expands the cellular scope for investigating the circadian rhythm–AF association: temporally organized signaling in the microvasculature, extracellular matrix, and immune–stromal compartments may contribute to the atrial pathogenic substrate captured by these candidate genes.

### Independent human and CREM mouse atria showed concordant transcript and protein increases

To evaluate whether the transcriptomic findings can be generalized across species and molecular layers, we quantified candidate RNAs and proteins in independent human tissue samples and atria of CREM mouse models. In the human cohort, CP and ANGPTL2 messenger RNA (mRNA) levels were significantly higher in the left atrial appendage tissue from patients with persistent AF than in that from patients in SR (Figures 8A,B), which reproduced the expression trend observed in the discovery analysis. In 28-week-old heterozygous male CREM mice, Cp and Angptl2 mRNA levels were higher in atrial tissue than in wild-type controls (Figures 8C,D). This cross-species consistency extends the observed candidate gene expression pattern to a well-established atrial arrhythmia model.

**FIGURE 8:**
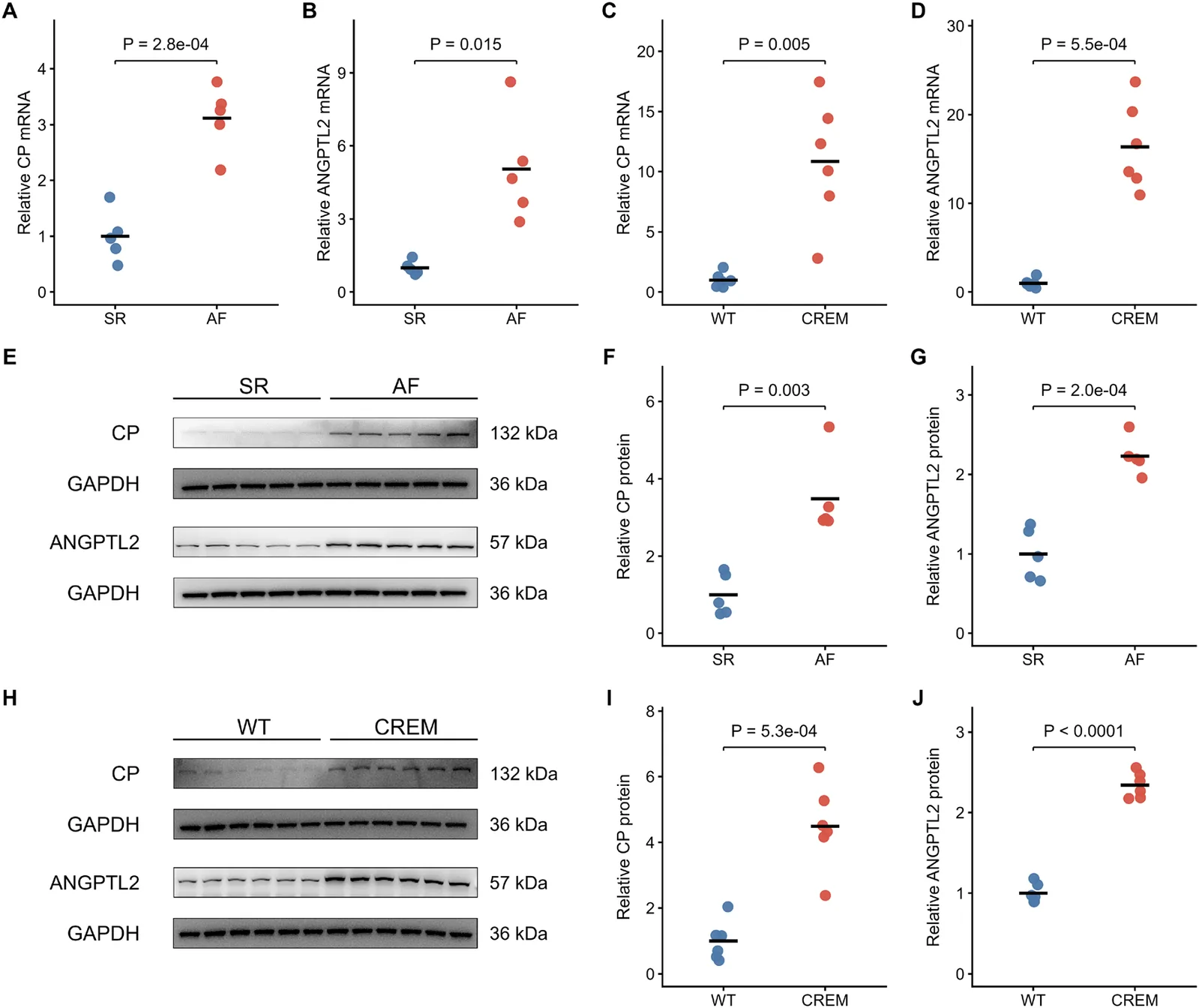
Experimental validation of CP and ANGPTL2 expression in human atrial tissue and CREM mice. **(A,B)** Human *CP* and *ANGPTL2* mRNA levels in sinus-rhythm and AF tissue. **(C,D)** Mouse *Cp* and *Angptl2* mRNA levels in wild-type and CREM atria. **(E–G)** Human immunoblots and protein quantification. **(H–J)** Mouse immunoblots and protein quantification. Both transcripts and proteins were higher in AF or CREM atria.

Human immunoblotting assays revealed higher protein abundance of CP and ANGPTL2 in AF tissue (Figures 8E–G). Consistent with this finding, immunoblotting of mouse samples detected the same direction of change in CREM atrial tissue (Figures 8H–J). Concordance between transcriptomic and proteomic measurements enhances the biological plausibility of our findings and supports a conserved remodeling-associated response. The persistence of consistent signals across both species and molecular layers indicates that these circadian-associated candidates mark a reproducible component of the remodeling-associated state, rather than platform-specific transcriptomic fluctuations. This observation provides a robust foundation for future time-resolved expression analysis and perturbation experiments.

Genotypic analysis confirmed the heterozygous CREM genotype, and representative limb-lead electrocardiographic recordings detected premature atrial contractions in the CREM model (Supplementary Figure S1). These observations link the molecular characterization to the arrhythmia-prone phenotype in our experimental model.

### Cross-cohort drug-reversal screening did not identify a compound suitable for advancement

To investigate whether a disease-associated transcriptional state can be used to identify candidates for pharmacological reversal, we constructed a cross-cohort consensus signature. Among the 15,711 genes detected in at least two cohorts, 313 satisfied the primary consensus definition, consisting of 137 upregulated genes and 176 downregulated genes. Fifty-nine of these genes satisfied the more stringent three-cohort definition. For subsequent drug-reversal screening, we utilized the top 50 ranked upregulated genes and the top 50 ranked downregulated genes.

The L1000FWD analysis identified 50 opposing signatures corresponding to 27 named compounds. Among these, SB 225002 was the only named compound with an adjusted P-value below the 0.05 significance threshold (adjusted P = 0.0487). SB 225002 had no independent reversal records in the CREEDS database and no confirmed direct ANGPTL2 or CP target-perturbation evidence in the iLINCS database. Among 875 curated drug signatures from CREEDS, including 450 human-derived signatures, none satisfied the prespecified independent reversal criterion. Furthermore, iLINCS contained no confirmed direct target records for ANGPTL2 or CP. For the short-term CP perturbation analysis, the returned results were text matches of compound names rather than verified gene-target records.

Consequently, none of the 27 prioritized candidate compounds met all combined selection criteria, and the primary screening was terminated. This negative result avoids the arbitrary prioritization of database-derived compounds and explicitly delineates the boundary between transcriptomic reversal screening and subsequent downstream molecular docking analysis. With respect to the circadian-associated candidates identified in the preceding analysis, the observation that no single perturbagen could robustly reverse the pan-cohort AF signature indicates that future screening frameworks should explicitly incorporate atrial cell type, compound exposure time, and circadian phase, rather than assuming that disease signatures are temporally invariant.

### Clinically used antiarrhythmic drugs met the exploratory docking threshold

Since none of the screen-identified compounds met the criteria for further development, we next investigated a separate question: whether three clinically used antiarrhythmic drugs exhibit predicted structural compatibility with ANGPTL2 or CP. Amiodarone, flecainide, and propafenone were individually docked to both target proteins (Supplementary Figures S3A–F). All six of the lowest-energy docking poses fell below the prespecified exploratory threshold of −5.0 kcal mol^-1^. For binding to CP, the docking scores were −8.0, −8.0, and −7.4 kcal mol^-1^ for amiodarone, flecainide, and propafenone, respectively. The corresponding scores for binding to ANGPTL2 were −6.6, −8.0, and −7.6 kcal mol^-1^ (Supplementary Figure S3G). The consistent satisfaction of the threshold criterion supports direct biophysical testing of these candidate–drug–protein pairs. In the context of our previous rhythmicity findings, these pairs also constitute a tractable experimental framework to investigate whether clinically used rhythm-control drugs interact with circadian-associated remodeling proteins and whether any such interaction varies with temporal cellular state. It should be noted that the docking results alone do not confirm direct binding, functional modulation of ANGPTL2 or CP, or antiarrhythmic efficacy mediated by either protein.

## Discussion

In this study, we used a staged evidence-based hierarchical framework to progress from circadian-associated transcriptomic discovery to external replication, temporal characterization, cellular localization confirmation, and experimental expression validation. The core finding of this study is that both ANGPTL2 and CP passed independent validation in the GSE41177 cohort, rather than remaining candidate genes supported only by discovery-cohort-based selection. Their external expression profiles were complementary rather than identical: ANGPTL2 exhibited stronger discriminatory power for distinguishing atrial fibrillation cases from controls, while CP showed a larger expression effect size and moderate discriminatory performance. Combined with the observed atrial rhythmicity, non-cardiomyocyte localization, and concordant expression patterns in human and mouse samples, this association links the two candidate genes to the overlapping inflammatory-metabolic and copper-redox pathways underlying atrial remodeling.

ANGPTL2 establishes the most definitive link between extrinsic stress and vascular, stromal, and inflammatory remodeling. Specifically, ANGPTL2 has been demonstrated to promote chronic inflammation, endothelial dysfunction, and impaired cardiac metabolism (Tabata et al., 2009; Horio et al., 2014; Tian et al., 2016). Accumulating evidence confirms that inflammatory signaling and immune cell recruitment are active drivers of AF substrate formation, rather than secondary byproducts of the pathological process (Ajoolabady et al., 2022; Dobrev et al., 2023; Grune et al., 2021; Hulsmans et al., 2023). The consistent upregulation of ANGPTL2 in the GSE41177 cohort and the AUC of 0.922 demonstrate that the ANGPTL2 signature is not restricted to the discovery cohort of patients with mitral regurgitation. Furthermore, the localization of ANGPTL2 to pericytes, endothelial cells, and fibroblasts supports the hypothesis that atrial ANGPTL2 reflects coordinated adaptive changes across non-cardiomyocyte compartments. This expression pattern positions ANGPTL2 as a candidate factor that links inflammatory cell recruitment, microvascular dysfunction, and extracellular matrix remodeling. Although the present findings cannot distinguish whether ANGPTL2 acts as an active pathogenic mediator or a passive state marker, they prioritize functional investigations targeting the vascular and stromal compartments for future research.

CP extends the range of replicated candidate biomarkers from inflammatory-metabolic remodeling to copper homeostasis, ferroxidase activity, and the oxidative stress microenvironment in AF (Kim et al., 2005; Liu et al., 2022; Yang et al., 2023b). Oxidative and metabolic stress synergistically promote atrial structural and electrical remodeling via mitochondrial, endoplasmic reticulum, and redox signaling pathways (Bode et al., 2024; Yuan et al., 2022). In the GSE41177 dataset, CP exhibited a 2.20-fold upregulation, which exceeded the prespecified validation threshold, and independently discriminated AF tissue from healthy controls with an AUC of 0.771. This external validation result, combined with elevated CP RNA and protein abundance in independent human atrial samples and the CREM model, supports that CP is a reproducible remodeling-associated biomarker rather than a context-dependent co-factor of ANGPTL2. The lower AUC observed, despite a larger magnitude of mean expression change, may reflect greater within-group expression heterogeneity, which is biologically plausible for an acute-phase and redox-responsive protein. Additionally, CP transcript levels do not reflect copper binding occupancy, ferroxidase catalytic activity, or post-translational oxidative modification. Therefore, successful transcript-level replication justifies further biochemical investigation of the copper-redox status in phenotypically and temporally well-resolved AF cohorts but does not support immediate clinical application as a diagnostic biomarker.

The circadian interpretation is supported by convergent evidence rather than by GeneCards membership alone. Cardiac circadian clocks regulate metabolism, contraction, electrophysiology, and stress-responsive transcription, and temporal disorganization of cardiac circadian rhythms has been linked to increased arrhythmia susceptibility (Durgan and Young, 2010; Fishbein et al., 2021; Hayter et al., 2021; Kelters et al., 2025; Ruan et al., 2021; Yang et al., 2023b). ANGPTL2 and CP also satisfy rhythmicity criteria in the GTEx heart atrial appendage cohort. This observation increases the possibility that biological sample collection time contributes to inter-individual variation in the observed atrial abundance of these two transcripts. This dataset does not allow us to conclude that AF alters circadian rhythm amplitude or phase because the collection time for each sample was not recorded and the GTEx study was not designed as an AF time-series experiment. Prospective data collection with time-stamped sampling is therefore required to rigorously evaluate whether AF modifies circadian expression amplitude, phase, or clock-dependent regulation of target genes.

Immune deconvolution, clustering, and single-nucleus analyses position the candidate genes within a broader multicellular remodeling microenvironment. The analyzed AF samples exhibited heterogeneous activity across monocyte, macrophage, B-cell, lysosomal, neutrophil, and muscle contraction gene programs, and the expression levels of candidate genes correlated with the inferred abundances of selected immune cell populations. The vascular and stromal localization of ANGPTL2 and CP is consistent with recent single-cell and single-nucleus studies that resolve AF-associated signals across both cardiomyocyte and noncardiomyocyte compartments (Hill et al., 2024; Leblanc et al., 2024; Selewa et al., 2023). These findings indicate that candidate-associated remodeling may involve intercellular crosstalk among endothelial cells, pericytes, fibroblasts, and immune cell populations. This defined cellular context provides biological interpretability to bulk-tissue omics signals, while the inferred inflammatory–contractile axis remains a testable hypothesis rather than a validated AF-specific taxonomy.

Our pharmacological analysis was structured as a sequential decision-making framework, rather than an unrestricted search for a single lead compound. Although connectivity map-based methods can associate disease signatures with drug-induced perturbation profiles, database-derived ranking alone is insufficient to confirm reproducible signature reversal and target engagement (Lamb et al., 2006; Subramanian et al., 2017). Our top-50 primary screening yielded 27 annotated compounds, none of which met the combined validation criteria from L1000FWD, CREEDS, and iLINCS (Wang et al., 2016; 2018; Pilarczyk et al., 2022). By retaining and reporting this negative result, we avoided drawing premature mechanistic conclusions for SB 225002 or other insufficiently validated candidate hits.

The subsequent docking analysis addressed a distinct, clinically relevant research question. Amiodarone, flecainide, and propafenone were selected because they are well-established rhythm-control therapeutics, not because they were identified using the reversal screen (Joglar et al., 2024). All six candidate–drug pairs met the exploratory threshold, with the lowest binding affinity score reaching −8.0 kcal mol^-1^. These results identify promising candidate–drug pairs for validation using surface plasmon resonance, microscale thermophoresis, cellular target-engagement assays, and functional perturbation experiments. However, it should be noted that docking scores rank binding poses within a computational model and are sensitive to multiple confounding factors, including receptor conformation, pocket definition, protonation state, and inherent scoring-function errors (Trott and Olson, 2010; Ferreira et al., 2015). Docking results only indicate structural plausibility and support for subsequent experimental investigation, rather than definitive evidence of direct target binding or a novel mechanism of antiarrhythmic action.

Several limitations constrain the generalizability of these interpretations. GSE115574 is a small discovery cohort of patients diagnosed with severe primary mitral regurgitation, and its selection criteria of nominal P < 0.05 and a 1.2-fold change threshold were over-permissive for candidate identification. GSE41177 provides independent support for our findings but only includes three control participants and 16 participants with AF, where paired tissue sampling contributed six control arrays and 32 AF arrays. The primary analysis treated each array as an independent tissue observation; therefore, effect size estimates from this analysis cannot be interpreted as being derived from 38 independent participants. The participant-blocked sensitivity analysis retained both core associations but utilized a different set of probes. A single external dataset is insufficient to establish broad transportability across populations or prospective diagnostic performance of the identified signature. The broad candidate list obtained from GeneCards includes indirect circadian rhythm associations, while GTEx provides rhythmicity data at the tissue level in non-AF samples rather than time-series expression data from AF tissues. Immune deconvolution only yields estimates of relative cell fractions, the candidate-defined gene clusters are of small size, and single-nucleus localization analysis did not include donor-stratified case–control testing. The drug repurposing screen was restricted to the top 50 signature genes and limited by the coverage and cell context representation of the three included databases; failure to identify a tractable compound does not confirm that the AF-associated transcriptional program is pharmacologically irreversible. The human validation cohort included only five participants per group, and the *in vivo* mouse experiment was limited to 28-week-old male individuals. Finally, concordant expression changes and threshold-passing docking scores do not establish gene-specific function, direct molecular binding, *in vivo* target engagement, or therapeutic efficacy.

In summary, this study identifies ANGPTL2 and CP as externally validated circadian-associated AF candidate drivers with complementary predictive performance and biological profiles. Their consistent convergence across the cohort, human atrial transcriptome rhythmicity, cell-type-specific expression localization, and conserved expression in both human and mouse models supports the existence of a functional interface between temporal atrial biology, noncardiomyocyte inflammatory-metabolic remodeling, and copper-redox homeostasis. The negative drug-reversal screening establishes a transparent filtering threshold, while molecular docking of clinically approved antiarrhythmic drugs generates experimentally testable candidate gene–drug pairs, without claiming confirmed pharmacological mediation of the observed effects. Prospective time-resolved sampling, donor-stratified single-cell analysis, quantitative biochemical CP activity assays, targeted functional perturbation, and direct molecular binding assays are required prior to clinical diagnostic or therapeutic translation of these findings.

## Data Availability

The original contributions presented in the study are included in the article/Supplementary Material, further inquiries can be directed to the corresponding authors.
