# Supplementary material for: ANGPTL2 and CP define complementary circadian-associated signals in atrial fibrillation: integrative transcriptomics and external validation
Source: Front Cell Dev Biol. 2026 Sep 21;14:1949794. doi: 10.3389/fcell.2026.1949794 (PMC13635327; doi:10.3389/fcell.2026.1949794)
Supplement: Supplementary file 1 [file DataSheet1.pdf]

Supplementary Material

ANGPTL2 and CP define complementary circadian-associated signals in atrial fibrillation: integrative transcriptomics and external validation

Supplementary Methods

Cross-cohort drug-reversal screening

Three independent gene expression datasets (GSE115574, GSE282504 and GSE69890) were analyzed to construct a cross-cohort atrial fibrillation (AF) signature for pharmacological reversal screening. To avoid overfitting and maintain analytical independence, the three datasets were not merged, and the latter two cohorts were not reused as external validation sets for ANGPTL2 or the circulating protein (CP) in the revised evidence chain. Genes were retained for subsequent analysis if they met the threshold criteria: $P < 0.05$ and absolute $\log_2$ fold change ≥ 0.263. Primary consensus genes were required to satisfy the filtering threshold with consistent expression directionality in at least two cohorts, show no significant expression change passing the threshold in the opposite direction in any cohort, and have a random-effects meta-analysis $P < 0.05$. The 50 top-ranked upregulated genes and the 50 top-ranked downregulated genes were then submitted to the L1000FWD platform. Next, named compounds identified from the initial screening were evaluated for reversal efficacy via the CREEDS and iLINCS databases. A compound was considered to have sufficient translational advancement potential only if it met all the following criteria: statistically significant FDR in L1000FWD, independent directionally consistent reversal of the AF signature in CREEDS, and available interpretable target or translational evidence.

Exploratory molecular docking of clinically used antiarrhythmic drugs

Amiodarone, flecainide, and propafenone were selected *a priori* because they are first-line pharmacological agents for rhythm control in patients with atrial fibrillation (AF) (Joglar et al., 2024). This selection was performed independently of the results from the prior negative drug-reversal screen. The X-ray crystal structures of human ceruloplasmin (CP, RCSB PDB: 1KCW; resolution = 3.0 Å) and the fibrinogen-like globe domain of human angiopoietin-like 2 (ANGPTL2, RCSB PDB: 6Y41; resolution = 1.79 Å) were used as docking receptors. The three-dimensional structures of amiodarone (PubChem CID: 2157), flecainide (PubChem CID: 3356), and propafenone (PubChem CID: 4932) were prepared using Chem3D v15.0, and both receptor and ligand files were converted to PDBQT format for subsequent analysis. Molecular docking was performed with AutoDock Vina v1.1.2.35 (Trott and Olson, 2010). For ANGPTL2, a 126 × 126 × 126 Å search box was applied; the two search box centers used across all ligand docking runs were (22.052, −32.621, −48.335) and (22.052, −36.767, −48.335), as reported in prior work. For CP, the three reported center/size configurations used for the search box were (136.410, 34.215, 2.023) with an 80 × 82 × 76 Å grid, (136.410, 32.240, 0.744) with an 82 × 76 × 80 Å grid, and (134.492, 34.215, 0.744) with a 74 × 84 × 82 Å grid. The energy range was set to 5 kcal mol^{-1}, the top 20 binding modes were retained, and the default exhaustiveness setting was used. The lowest-energy predicted binding pose was retained for visualization in PyMOL v3.0.4 (Ferreira et al., 2015). A docking score below −5.0 kcal mol^{-1} was used exclusively as an exploratory threshold for pose ranking, and was not interpreted as experimental evidence of target binding.

Discovery-threshold sensitivity analysis

The discovery limma analysis results were re-evaluated at absolute $\log_2$ fold-change thresholds of 0.263, 0.5, and 1.0, using both the nominal $P < 0.05$ and Benjamini-Hochberg false discovery rate (BH FDR) < 0.05 significance cut-offs. For ANGPTL2, the $\log_2$ fold change was 0.851, nominal $P = 2.87 \times 10^{-5}$, and FDR = 0.090; for CP, the $\log_2$ fold change was 0.415, nominal P = 0.00317, and FDR = 0.477. As the effect-size threshold increased from 0.263 to 0.5 and 1.0, the number of DEGs identified by nominal P gradually decreased from 479 to 129 and 19, respectively.

GSE41177 paired-tissue sensitivity analysis

The primary analysis of the GSE41177 dataset replicated the published tissue array workflow using pre-specified probes. Because left atrial appendage tissues and left atrial-pulmonary vein junction tissues were paired within individual participants, we conducted a sensitivity analysis using the `limma` package's `duplicateCorrelation` method with participant as the blocking factor. The sensitivity analysis workflow selected the best-annotated target probe under the paired-tissue model: 213001_at for *ANGPTL2* and 204846_at for *CP*. These sensitivity estimates are reported separately from the primary pre-specified probe results and were not pooled across different probe selection specifications.

Supplementary Results

External replication in GSE41177

In the primary exact-reproduction analysis, the expression of ANGPTL2 probe 219514_at was significantly upregulated in atrial fibrillation (AF) tissue (log2 fold change = 0.760; Welch's *P* = 6.84 × 10^{-9}; FDR = 1.37 × 10^{-8}), achieving an area under the receiver operating characteristic curve (AUC) of 0.922 (95% CI: 0.832-1.000). The expression of CP probe 1558034_s_at exhibited a larger magnitude of expression change (log2 fold change = 1.137; Welch's *P* = 0.0342; FDR = 0.0342) with an AUC of 0.771 (95% CI: 0.592-0.950). Both candidate genes satisfied the prespecified combined inclusion criterion that the absolute log2 fold change is at least 0.263 and the unadjusted *P* value is less than 0.05. In the participant-blocked sensitivity analysis, ANGPTL2 remained significantly upregulated when assessed with probe 213001_at (log2 fold change = 1.397; *P* = 3.32 × 10^{-6}; FDR = 1.94 × 10^{-4}), and CP remained significantly upregulated when assessed with probe 204846_at (log2 fold change = 1.416; *P* = 0.0101; FDR = 0.0196). Therefore, both genes retained directional consistency and statistical support after modeling within-participant tissue pairing, even though probe-specific expression estimates are not directly comparable.

Cross-cohort drug-reversal screening

Among 15,711 genes detected in at least two cohorts, 313 satisfied the primary consensus definition (137 upregulated and 176 downregulated), and 59 satisfied the strict three-cohort definition (25 upregulated and 34 downregulated). L1000FWD identified 50 opposing signatures corresponding to 27 named compounds. Only SB 225002 had an adjusted P-value < 0.05 (adjusted P-value = 0.0487), but it lacked independent CREEDS reversal support as well as direct evidence of perturbation for ANGPTL2 or CP in the iLINCS database. None of the 875 CREEDS drug signatures, including 450 human-derived signatures, satisfied the predefined reversal criterion. The iLINCS database contained no confirmed target records for ANGPTL2 or ceruloplasmin. Consequently, all 27 named compounds were classified as unsuitable for further progression, and no screen-identified compound proceeded to molecular docking.

Exploratory docking of clinically used antiarrhythmic drugs

Amiodarone, flecainide, and propafenone were selected independent of the reverse screening because they are clinically approved antiarrhythmic agents. Their binding scores against CP were −8.0, −8.0, and −7.4 kcal mol^{-1}, respectively; and the corresponding binding scores against ANGPTL2 were −6.6, −8.0, and −7.6 kcal mol^{-1}. All six values exceeded the exploratory pose-ranking threshold of −5.0 kcal mol^{-1}. These results only indicate binding compatibility within the scope of the specified docking boxes and scoring function. They do not confirm experimental binding, target engagement, target-mediated expression regulation, antiarrhythmic activity mediated by ANGPTL2 or CP, or valid drug repurposing indications.

Supplementary Table 1 | Quantitative PCR primer sequences

Target	Species	Direction	Sequence (5-prime to 3-prime)
GAPDH	Human	Forward	CTGGGCTACACTGAGCACC
GAPDH	Human	Reverse	AAGTGGTCGTTGAGGGCAATG
Gapdh	Mouse	Forward	AGGTCGGTGTGAACGGATTTG
Gapdh	Mouse	Reverse	GGGGTCGTTGATGGCAACA
CP	Human	Forward	GGGCCATCTACCCTGATAACA
CP	Human	Reverse	TTAAAGGTCCGATGAGTCCTGA
Cp	Mouse	Forward	CTTAGCCTTGGCAAGAGATAAGC
Cp	Mouse	Reverse	GATGACAGGGCCTAAAAACCC
ANGPTL2	Human	Forward	GAACCGAGTGCATAAGCAGGA
ANGPTL2	Human	Reverse	GTGACCCGCGAGTTCATGTT
Angptl2	Mouse	Forward	CCACCTCGGGTCTACCAAC
Angptl2	Mouse	Reverse	CTTGCAGGCAGTCTCTCCAT

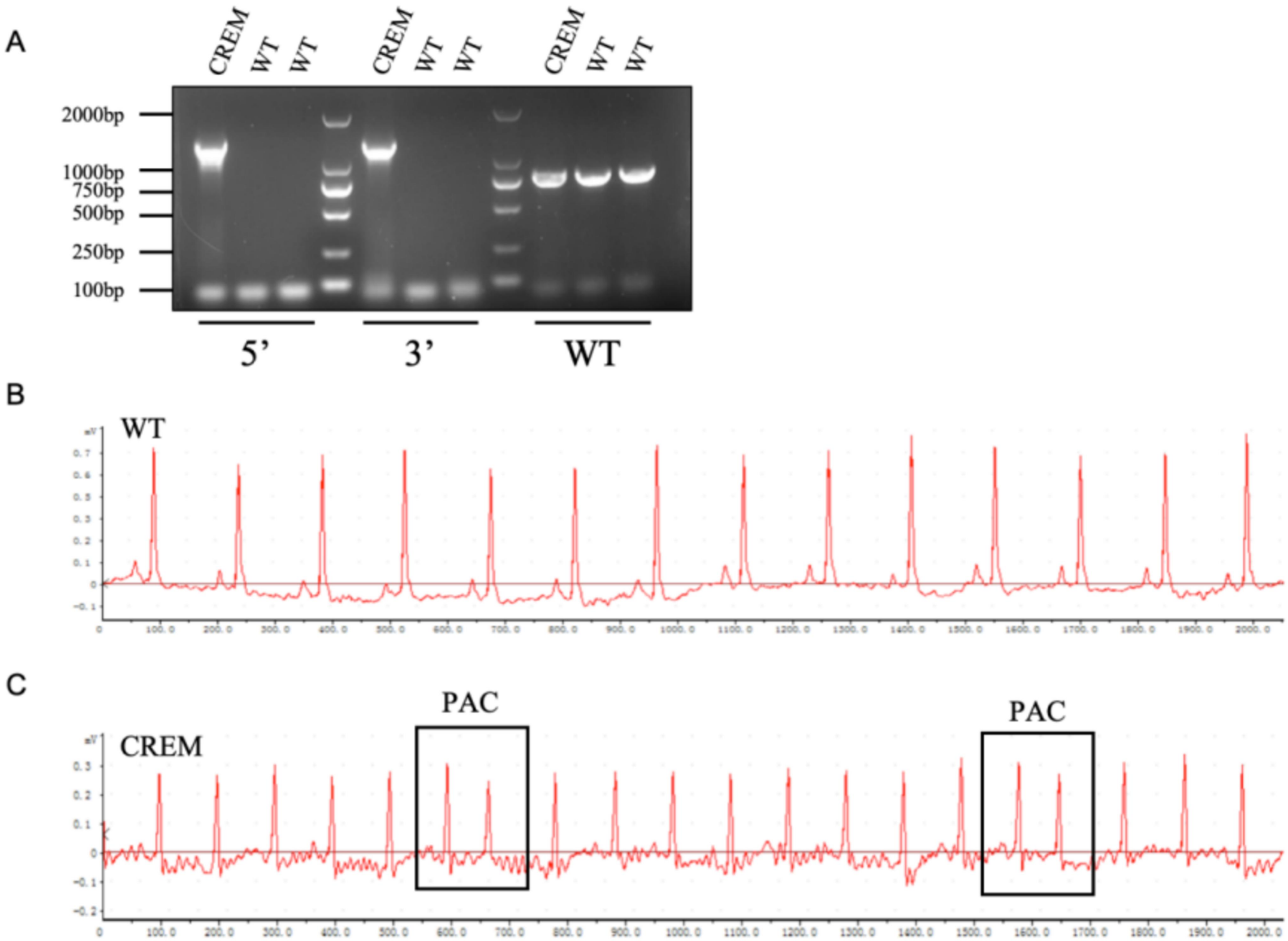

Supplementary Figure 1. PCR genotyping and representative electrocardiograms of wild-type (WT) and CREM mice. (A) Genotyping with 5′-arm, 3′-arm and WT primer sets. (B,C) Limb-lead recordings from 28-week-old male WT and heterozygous CREM mice. Boxes mark premature atrial contractions (PACs) in the CREM trace.

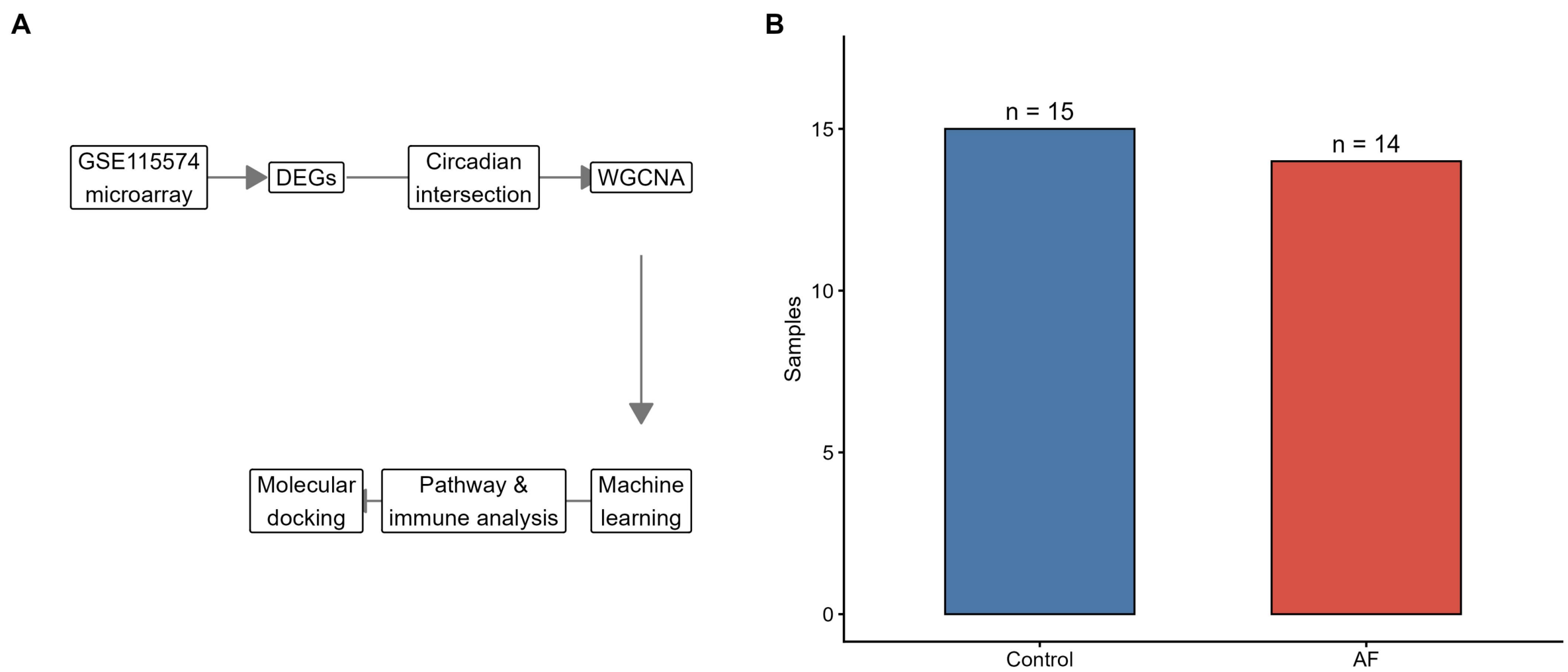

Supplementary Figure 2. Study workflow and discovery-cohort composition. (A) Schematic of the discovery and candidate-prioritization workflow. (B) Numbers of control and AF samples in GSE115574.

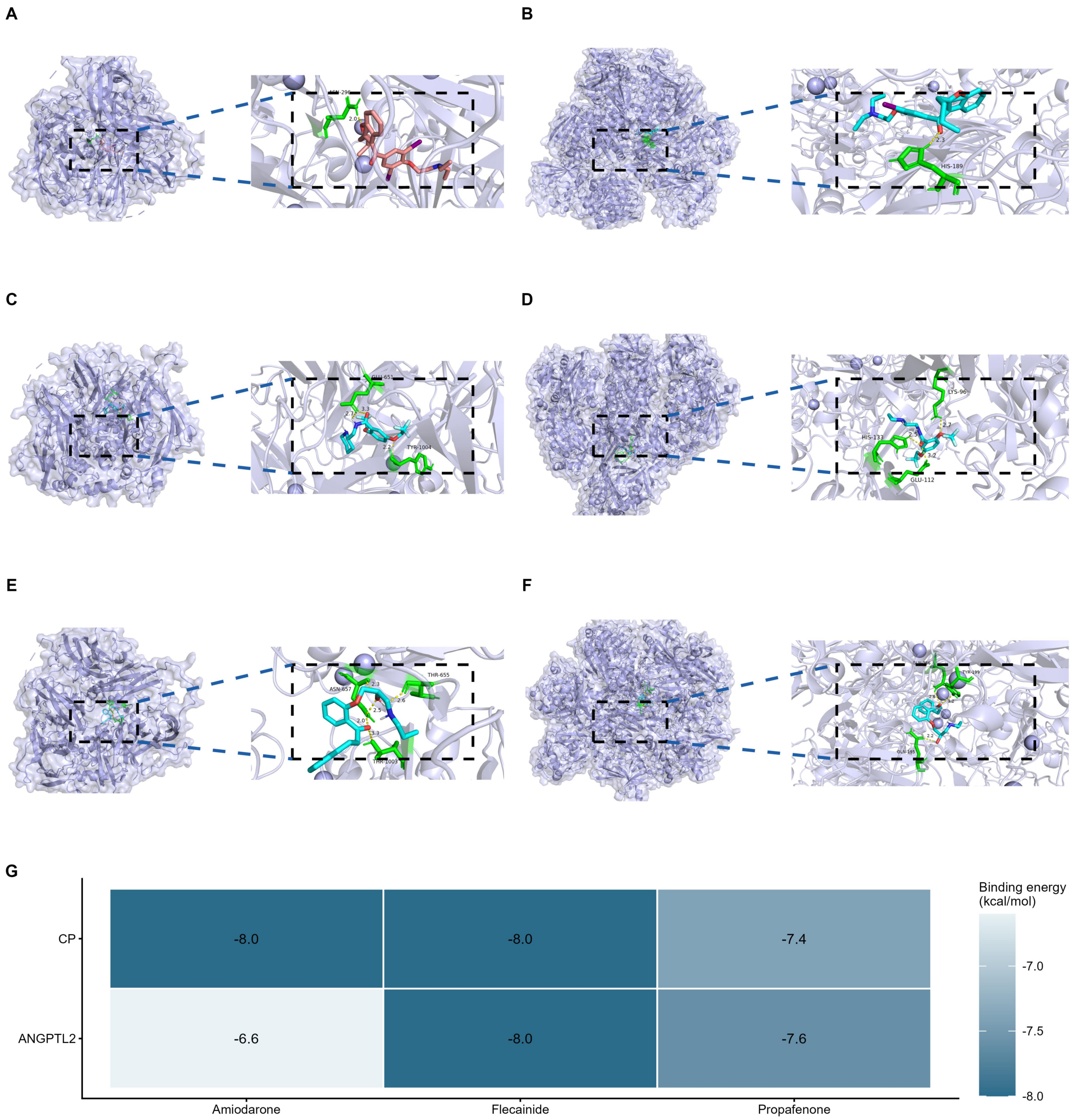

Supplementary Figure 3. Exploratory molecular docking of clinically used antiarrhythmic drugs with *ANGPTL2* and *CP*. Amiodarone, flecainide and propafenone were selected independently of the negative drug-reversal screen. (A-F) Predicted poses of the three drugs with *ANGPTL2* or *CP*. (G) Heatmap of docking scores. Docking results indicate predicted pose compatibility only.
